# Special Issue “Genetic, Functional and Therapeutic Aspects of Procoagulant and Anticoagulant Factors”

**DOI:** 10.3390/ijms25115741

**Published:** 2024-05-25

**Authors:** Tami Livnat, Rima Dardik

**Affiliations:** The Amalia Biron Research Institute of Thrombosis and Hemostasis, Faculty of Medical and Health Sciences, Tel-Aviv University and the National Hemophilia Center Sheba Medical Center, Tel-Hashomer 52621, Israel

Pro- and anticoagulant factors are core components of hemostasis. The foundational exploration of hemostasis dates back several centuries, with significant advancements in the understanding of blood coagulation made between the 19th and early 20th centuries.

In 1905, a key publication by Paul Morawitz, titled “Die Chemie der Blutgerinnung” (The Chemistry of Blood Coagulation), was the first to systematically describe the coagulation cascade. In this seminal work, Morawitz assembled the available data of the time and proposed a “Classical Theory” of blood coagulation. This work was groundbreaking, as it detailed the interactions of what we now refer to as fibrinogen, prothrombin, thrombin, and calcium ions in the coagulation cascade [1].

Later on, the understanding of hemostasis evolved into a complex cascade model, distinguishing between primary and secondary phases and highlighting the importance of platelets in hemostasis [2]. Primary hemostasis begins with the rapid formation of a platelet plug at the site of vascular injury, where activated platelets adhere to the damaged endothelium and aggregate to form a temporary seal. This initial response is swiftly followed by secondary hemostasis, which involves the waterfall sequence activation of coagulation factors, leading to a stable, cross-linked mesh that ensures a stable clot [3]. This complex interplay between platelet actions and the coagulation cascade is crucial for effective hemostasis.

Building on this established foundation, the field of hemostasis research has further evolved to include a sophisticated “cell-based model” of coagulation [4]. This model elucidates the interactions between cellular components and coagulation factors, introducing an integrated approach to understanding clot formation. This cell-based model is structured around three key phases: initiation, which occurs on tissue factor-bearing cells and results in the generation of a small but pivotal amount of thrombin; amplification, where this thrombin activates additional platelets and coagulation factors on the platelet surface, enhancing the coagulation response; and propagation, during which the activated platelets serve as a site for significant thrombin production, leading to the formation of a robust and stable clot. This advanced model emphasizes the dynamic and regulated nature of hemostasis, highlighting how cellular interactions are critical to the effective functioning of the coagulation pathways. It not only offers a more comprehensive perspective on the coagulation process but also opens new avenues for targeted therapeutic strategies that address specific stages of the hemostatic process.

The “termination phase” of the coagulation cascade plays a critical role in preventing excessive clot formation and limiting clotting to the injury site. Natural anticoagulant pathways such as the protein C system are activated by thrombin bound to thrombomodulin on endothelial cells, which leads to the inactivation of factors Va and VIIIa. Additionally, antithrombin III inhibits thrombin and other serine proteases, while the fibrinolytic system initiates the degradation of the fibrin meshwork through plasmin, resolving the clot and restoring vascular integrity [5]. These mechanisms ensure the self-limiting and controlled process of hemostasis, effectively balancing clot formation and resolution to maintain vascular health and prevent pathological thrombosis.

Moreover, Rudolf Virchow’s pioneering work highlighted the importance of the vascular system’s integrity in thrombosis prevention. “Virchow’s triad”, including endothelial injury, stasis, and hypercoagulability, is fundamental in determining preventative strategies and treatments for thrombosis and vascular diseases [6].

The broader implications of coagulation extend beyond the vascular system, affecting inflammatory processes, wound healing, and tumor progression [7,8]. This underscores the need for a comprehensive understanding of hemostasis mechanisms to develop therapeutic strategies and interventions.

The therapeutic implications of research into coagulation factors are profound. With advancements in genetic engineering and drug design, targeted therapies that correct specific coagulation factor deficiencies or malfunctions are becoming feasible. This approach not only promises more effective management of disorders such as hemophilia and thrombophilia but also minimizes the adverse effects associated with traditional treatments. Moreover, recent developments in oral anticoagulants have significantly expanded therapeutic options, offering safer and more convenient alternatives for the long-term management of thrombotic disorders, enhancing patient compliance, and improving outcomes.

This Special Issue therefore assembles a rich array of research that expands our understanding of procoagulant and anticoagulant factors across the broad spectrum of hemostasis. The studies published in this Special Issue address the intricate balance between anti-inflammatory and antithrombotic therapies in managing acute cardiovascular events, the impact of enhanced hypercoagulability on the outcomes of patients with infectious diseases, and the role of platelets in hemostasis, including the molecular mechanisms regulating platelet receptor function and expression in various clinical settings, in addition to their involvement in bleeding complications in surgical patients.

Additionally, various therapeutic strategies are presented, including novel thrombin receptor antagonists and genetically engineered activated protein C, offering promising avenues for modulating inflammation.

The therapeutic options currently available for hemophilia A patients mainly include recombinant factor VIII concentrates [9] and gene therapy based on the delivery of a functional F8 gene into the liver using adeno-associated viral vectors [10]. A review contributed by Sarafanov presents strategies for favorable molecular modifications of FVIII to increase protein expression, potentially improving the therapeutic efficacy of both FVIII replacement products and constructs used in hemophilia A gene therapy [11]. Lastly, this Special Issue presents various aspects of the genetic diagnosis of antithrombin deficiency and hemophilia A, highlighting both historical evolution and future challenges [12]. Collectively, these studies emphasize the importance of continued research and innovation in the treatment of coagulation disorders, based on a comprehensive understanding of the molecular and functional mechanisms of hemostasis in order to develop effective therapies and improve patient outcomes.
